# An exploration of the genetic epidemiology of non-suicidal self-harm and suicide attempt

**DOI:** 10.1186/s12888-021-03216-z

**Published:** 2021-04-23

**Authors:** Abigail Emma Russell, Gibran Hemani, Hannah J Jones, Tamsin Ford, David Gunnell, Jon Heron, Carol Joinson, Paul Moran, Caroline Relton, Matthew Suderman, Sarah Watkins, Becky Mars

**Affiliations:** 1 Children and Young People’s Mental Health Research Collaboration (ChYMe), University of Exeter College of Medicine and Health, Exeter, UK; 2 MRC Integrative Epidemiology Unit, University of Bristol Medical School, Bristol, UK; 3 Population Health Sciences, University of Bristol Medical School, Bristol, UK; 4 Centre for Academic Mental Health, Population Health Sciences, University of Bristol Medical School, Bristol, UK; 5 University of Cambridge Department of Psychiatry, Cambridge, UK; 6 NIHR Biomedical Research Centre at the University Hospitals Bristol NHS Foundation Trust and the University of Bristol, Bristol, UK

**Keywords:** Suicide, self-harm, non-suicidal self-injury, Genetic epidemiology, Polygenic risk scores

## Abstract

**Background:**

Empirical evidence supporting the distinction between suicide attempt (SA) and non-suicidal self-harm (NSSH) is lacking. Although NSSH is a risk factor for SA, we do not currently know whether these behaviours lie on a continuum of severity, or whether they are discrete outcomes with different aetiologies. We conducted this exploratory genetic epidemiology study to investigate this issue further.

**Methods:**

We explored the extent of genetic overlap between NSSH and SA in a large, richly-phenotyped cohort (the Avon Longitudinal Study of Parents and Children; *N* = 4959), utilising individual-level genetic and phenotypic data to conduct analyses of genome-wide complex traits and polygenic risk scores (PRS).

**Results:**

The single nucleotide polymorphism heritability of NSSH was estimated to be 13% (SE 0.07) and that of SA to be 0% (SE 0.07). Of the traits investigated, NSSH was most strongly correlated with higher IQ (rG = 0.31, SE = 0.22), there was little evidence of high genetic correlation between NSSH and SA (rG = − 0.1, SE = 0.54), likely due to the low heritability estimate for SA. The PRS for depression differentiated between those with NSSH and SA in multinomial regression. The optimal PRS prediction model for SA (Nagelkerke *R*^2^ 0.022, *p* < 0.001) included ADHD, depression, income, anorexia and neuroticism and explained more variance than the optimal prediction model for NSSH (Nagelkerke R^2^ 0.010, *p* < 0.001) which included ADHD, alcohol consumption, autism spectrum conditions, depression, IQ, neuroticism and suicide attempt.

**Conclusions:**

Our findings suggest that SA does not have a large genetic component, and that although NSSH and SA are not discrete outcomes there appears to be little genetic overlap between the two. The relatively small sample size and resulting low heritability estimate for SA was a limitation of the study. Combined with low heritability estimates, this implies that family or population structures in SA GWASs may contribute to signals detected.

**Supplementary Information:**

The online version contains supplementary material available at 10.1186/s12888-021-03216-z.

## Background

Self-harm is a major public health concern. It is the strongest predictor of completed suicide and is particularly common among young people; a group in which rates of serious self-harm appear to be rising [1]. Genome-wide association studies (GWASs) have advanced our understanding of the genetic architecture of many complex traits. Yet, in comparison to other psychiatric traits [2–5], the genetic epidemiology of suicide and self-harm remains poorly understood. Whilst emerging evidence from twin studies estimate heritability of 17–55% for suicide and suicide attempts (SA) [6, 7], single nucleotide polymorphism (SNP)-based heritability estimates of SA are much smaller at 2–6% [8, 9].

Understanding the genetic basis of suicide is complicated by the broad spectrum of behaviours that fall under “suicidal behaviour”, which include self-harm with varying levels of suicidal intent as well as death by suicide. Within this broad definition there is a lack of consensus as to whether self-harm without suicidal intent (referred to as ‘non suicidal self-harm’ (NSSH)) and SA lie on a continuum of increasingly severe and lethal behaviour, or whether they should be considered discrete [10, 11]. This debate has been fuelled by the addition of both ‘non-suicidal self-injury’ and ‘suicidal behaviour disorder’ in the DSM-5 as conditions for further study [10, 12]. The existing observational evidence suggests that NSSH and SA share some risk factors, whilst others may be unique to one type of behaviour [13, 14]. However, disentangling the aetiology of these behaviours using observational data is challenging due to unmeasured and residual confounding [15]. Utilising genetic data to explore relationships between risk factors and suicidal behaviour reduces the potential for confounding and allows for a more nuanced exploration of traits that may confer liability to NSSH and/or SA.

Polygenic risk scores (PRS) utilise information from thousands of genetic variants to characterise a given individual’s genetic risk for a trait of interest [16, 17]. A recent study exploring risk for self-harm using the UK BioBank, a large well-characterised sample of middle-aged adults, found evidence of association between self-harm and PRSs for depression, schizophrenia, ADHD, bipolar disorder, alcohol use, and cannabis use, but no evidence for differential prediction of NSSH and SA [18]. A further study also utilising UK BioBank found positive genetic correlations of SA with neuroticism, schizophrenia and major depressive disorder (MDD) [8]. However, UK Biobank is socially-advantaged [19], and reports of lifetime self-harm and suicide attempt are very low compared with longitudinal cohorts [8, 13]. As such, further studies are needed to investigate whether these findings are generalisable to other samples.

This study uses data from the Avon Longitudinal Study of Parents and Children (ALSPAC) birth cohort to explore the genetic architecture and overlap of NSSH and SA, utilising individual-level genetic and phenotypic data to conduct genome-wide complex traits analysis and PRS prediction. Specifically, we aimed to:

1. Explore the SNP-based heritability of NSSH and SA, as well as other related psychological traits and sociodemographic risk factors previously associated with NSSH or SA.
2. Assess the genetic correlation between these related exposures, NSSH and SA.
3. Explore whether PRS for psychological and sociodemographic traits differentially predict NSSH and SA.

## Methods

### Sample

ALSPAC recruited pregnant women resident in Avon, UK with expected delivery dates between 1st April 1991 and 31st December 1992. The initial number of pregnancies enrolled was 14,541. Of these, 13,988 children were alive at 1 year of age [20–22], and genotype data were available for 8237. Data were collected via regular questionnaires and research clinics [23, 24]. Details of all data is available through a fully searchable data dictionary and variable search tool (http://www.bristol.ac.uk/alspac/researchers/our-data/). Ethical approval for the study was obtained from the ALSPAC Ethics and Law Committee and the Local Research Ethics Committees, and consent for biological samples has been collected in accordance with the Human Tissue Act (2004).

ALSPAC children were genotyped using the Illumina HumanHap550 quad chip genotyping platforms by 23andMe subcontracting the Wellcome Trust Sanger Institute, Cambridge, UK and the Laboratory Corporation of America, Burlington, NC, US. Following quality control assessment and imputation (see Supplementary Methods), genetic data was available for 8237 ALSPAC individuals.

### Non-suicidal self-harm and suicide attempt

ALSPAC participants reported at ages 16, 21, and 24 years whether they had “ever hurt [them]self on purpose in any way (e.g. by taking an overdose of pills, or by cutting [themselves])”. They were then asked if they “have ever seriously wanted to kill themselves on any occasion where they have hurt themselves” or whether the “last time they hurt themselves it was because they wanted to die”. Four thousand nine hundred and fifty-nine individuals had data from at least one time point. Responses were used to categorise participants into three groups: NSSH (17.0%, yes to self-harm, never reported intent to die), SA (9.5%, yes to self-harm, reported intent to die on at least one occasion), or no self-harm (73.5%). Three separate outcomes were utilised in the analyses: NSSH vs all others, SA vs all others, and a three-level outcome (no self-harm NSSH and SA). In addition, a further measure of SA was derived using an additional wave of ALSPAC data at age 26 years to match the phenotype used within a recent SA GWAS [9] .

### Genome-wide association study of non-suicidal self-harm and suicide attempt

We conducted a GWAS of NSSH and SA in ALSPAC using *snptest,* adjusting for age, sex and population substructure by including the first ten principle components. Results were filtered based on minor allele frequency of > 0.01 and an imputation quality (info) score of > 0.3.

### Estimating SNP heritability

Genome-wide complex traits analysis (GCTA) [25] was implemented to investigate SNP heritability (h^2^_SNP_) or the proportion of the variance of each phenotype explained by all observed SNPs for NSSH, SA and the 17 exposure phenotypes. A genetic relatedness matrix was calculated from Hapmap3 SNPs in unrelated child participants of ALSPAC. The heritability of each trait was estimated using restricted maximum likelihood analysis (REML), adjusting for the first 10 principal components and sex as covariates to mitigate confounding from population stratification. A complementary method of assessing SNP heritability is linkage disequilibrium score regression (LDSC) [26], which we also applied to GWAS results of NSSH and SA in ALSPAC.

### Genetic correlations

We aimed to assess genetic correlations (rG) between NSSH, SA and related phenotypes using bivariate REML [27].

### Polygenic risk score analyses

PRS were derived in ALSPAC for phenotypes shown to relate to NSSH/SA utilising external GWAS summary data, none of which contained ALSPAC participants. These phenotypes included psychiatric disorders, personality traits, sociodemographic and lifestyle characteristics (Table 1). Permission to use results from the MDD and personality GWASs [2, 32] was acquired from 23andMe.

**Table 1:** Related phenotype definitions in ALSPAC and other genome-wide association study data

Phenotype	GWAS details (for calculating PRS)					ALSPAC details (derivation of phenotypes)	
	Author, year	Total n	N cases	N controls	Original definition	Definition	Prevalence/mean (SD)
Depression	Howard (2019) [28]	807,553	246,363	561,190	Major depression- diagnostic interview/medical records/self-reported diagnosis or treatment for clinical depression	SMFQ > = 11, DAWBA depression at 7, 13 or 15, 17 years, self-report of ever being diagnosed with depression at 22	30%
Schizophrenia	Pardinas (2018) [4]	105,318	40,675	64,643	cases diagnosed	Self-reported “ever diagnosed with schizophrenia” asked at age 22	*N* = < 5
ADHD	Demontis (2019) [3]	40,366	20,183	20,183	cases diagnosed	DAWBA diagnosis at 7, 10, 13 or 15	3.31%
Autism Spectrum Disorders (ASC)	Grove (2017) [5]	46,351	18,382	27,969	registry-based cases	Mother report of Autism Spectrum Disorder diagnosis at age 7, in 2.5th percentile on autism traits at 9 years old, report of additional education or work support for autism or Asperger’s	2.46%
Suicide attempt	Erlangsen (2018) [9]	50,264	6024	44,240	Clinically-registered suicide attempts in psychiatric cases	suicide attempt ever by age 26- derived as for suicide attempt above plus self-reported “attempted suicide in past 12 months” at age 26 in Life Events questionnaire	10.31% (an additional 131 individuals over the age 24 variable)
Anxiety	Otowa (2016) [29]	21,761	7016	14,745	Standardised assessment instruments were used to generate DSM-based Anxiety disorder diagnoses, with some exceptions	Any anxiety disorder by DAWBA, age 7, 10, 13, 15 17, 24. Age 21 GAD7 > =10	14.30%
Problematic cannabis use	Demontis (2019) [30]	51,372	2387	48,985	Clinical records of ICD-10 codes for cannabis dependence	4+ score on CAST age 17 and 21	1.70%
Alcohol consumption	Clarke (2017) [31]	112,117			Self-reported average intake of alcohol consumption in units per week.	Average number of units per week based on frequency and number of drinks self-reported age 22 and 24	mean 2.83, SD 3.19
Openness	Lo (2016) [32]	76,551			Big-5, NEO Five-factor inventory and other validated personality measures	Big-5 questionnaire age 13	mean 35.8, SD 5.65
Conscientiousness	Lo (2016) [32]	59,225			Big-5, NEO Five-factor inventory and other validated personality measures	Big-5 questionnaire age 13	mean 31.9, SD 5.82
Extraversion	Lo (2016) meta-analysed with GPC-1 (as GPC-2 contains ALSPAC) [32]	76,600			Big-5, NEO Five-factor inventory and other validated personality measures	Big-5 questionnaire age 13	mean 35.3, SD 6.87
Agreeableness	Lo (2016) [32]	76,551			Big-5, NEO Five-factor inventory and other validated personality measures	Big-5 questionnaire age 13	mean 37.9, SD 5.19
Neuroticism	Nagel (2018) [33]	508,690			Big-5, NEO Five-factor inventory and other validated personality measures	Big-5 questionnaire age 13	mean 28.4, SD 6.58
Anorexia	Watson et al. (2019) [34]	72,517	16,992	55,525	Case definitions established a lifetime diagnosis of anorexia nervosa via hospital or register records, structured clinical interviews, or online questionnaires based on standardised criteria. In the UK Biobank, cases self-reported a diagnosis of anorexia nervosa	age 14, 16, 18, 24 range of questions regarding BMI, fasting, excessive exercise as in [35]	0.87%
IQ	Savage (2018) [36]	269,867			Different measures of intelligence were assessed in each cohort but were all operationalized to index a common latent g factor underlying multiple dimensions of cognitive functioning	WISC at age 8 and WASI at age 15	mean 96.31, SD 14.45
Educational attainment	Davies (2016) [37]	111,114			Binary education variable indexing whether or not each participant had self-reported attaining a college or university-level degree.	Binary- whether report graduating from University between age 21 and 24	47%
Household income	Hill (2016) [38]	112,151			Self-reported using a 5 point scale corresponding to the total household income before tax, 1 being less than £18,000, 2 being £18,000 - £29,999, 3 being £30,000 - £51,999, 4 being £52,000 – £100,000, and 5 being greater than £100,000	Average take-home income at age 22 and/or 23, split into 5 bands	mean 2.91, SD 1.00

*GPC* Genetics of Personality Consortium, *PRS* polygenic risk score, *GWAS* genome-wide association study, *ALSPAC* Avon Longitudinal Study of Parents and Children

We used *plink* to calculate PRS as the sum of the number of risk alleles a participant has (those with *p*-values below a threshold), weighted by their effect size [16]. PRS were created meeting a range of thresholds (5 × 10^− 8^, 1 × 10^− 7^, 1 × 10^− 6^, 1 × 10^− 5^, 1 × 10^− 4^, 0.001, 0.01, 0.05, 0.1, 0.2, 0.3, 0.4, 0.5) and standardised (mean of 0 and standard deviation of 1) prior to analyses. In order to determine the most appropriate threshold for each PRS, the associations between scores at each *p*-value threshold and ALSPAC phenotypes that matched as closely as possible to GWAS traits of interest (Table 1) were evaluated in regression models. The model with the largest *R*^2^ or Nagelkerke *R*^2^ for each phenotype was considered optimal, and the PRS at this threshold selected for the analyses.

A series of multinomial logistic regressions were first used to examine the association between each exposure PRS and a three-level outcome of no self-harm, NSSH and SA. The mean and variance of the self-harm outcome measure were similar (0.36 and 0.42 respectively), combined with results of goodness of fit tests this indicated that a Poisson regression model was suitable, with overdispersion not being a significant issue (*p* > 0.05). We then applied k-fold cross-validation lasso regression to ascertain the combination of polygenic scores that best predicted i) NSSH (vs all others) and ii) SA (vs all others). Ten folds were specified: for each iteration 90% of the data were used as the training sample and 10% as the prediction sample. Given the relatively small number of SA cases, we repeated the predication model for SA in post-hoc analyses using varying numbers of folds (k = 2–6). Results were consistent with the 10-fold model. Optimal predictive models for NSSH and SA were thus identified. For SA, we assessed whether the optimal prediction model accounted for more variance than using the PRS for SA derived from the external GWAS. No external PRS was available for NSSH for comparison. Analyses were conducted in Stata v15.

## Results

### SNP heritability

Sample sizes with both phenotypic and genetic data ranged from 1909 (cannabis use) to 7794 (Autistic Spectrum Conditions (ASC)). NSSH was estimated to have a h^2^_SNP_ of 0.132 (SE 0.07): 13.2% of the variance in NSSH was explained by common genetic variants (Table 2). In contrast, SA by age 24 years had negligible SNP heritability, and a large standard error relative to the estimate (h^2^_SNP_ 0.000, SE 0.07) indicating greater uncertainty in the estimation of heritability for SA. When extending the SA phenotype to age 26 years, we found a h^2^_SNP_ of 0.064 (SE 0.07). Heritability estimates from LDSC were consistent but with lower precision (Table 2).

**Table 2:** Estimated SNP heritability of psychiatric traits, personality and sociodemographic factors in ALSPAC using restricted maximum likelihood analysis and linkage disequilibrium score regression

Trait	n	% cases	h^**2**^_**SNP**_	SE	Log likelihood
	**Estimates from REML**				
Non-suicidal self-harm (main outcome)	4506	**17.31**	**0.13**	0.07	2174
Suicide attempt (main outcome)	4506	**9.25**	**0.00**	0.07	3345
ADHD	6440	**3.14**	**0.01**	0.05	8050
Alcohol consumption	3487		**0.18**	0.09	− 5760
Anorexia	5624	**1.03**	**0.00**	0.06	10,064
Anxiety	6644	**15.12**	**0.04**	0.05	3548
ASC	7794	**2.56**	**0.00**	0.04	10,434
Cannabis problematic use	1909	**1.78**	**0.00**	0.16	2891
Conscientiousness	4121		**0.15**	0.08	− 9272
Depression	6742	**31.50**	**0.01**	0.05	1940
Education	4007	**49.45**	**0.38**	0.08	784
Extraversion	4311		**0.10**	0.08	−10,407
Income	2929		**0.00**	0.11	− 1397
IQ	5672		**0.37**	0.06	−17,900
Neuroticism	4184		**0.03**	0.08	− 9818
Agreeableness	4239		**0.14**	0.08	− 8802
Schizophrenia	2744	**< 0.50%**	**0.14**	0.12	9446
Openness	4223		**0.23**	0.08	− 9360
Suicide attempt (by age 26)	4739	**10.49**	**0.06**	0.07	3249
	**Estimates from LDSC**				
NSSH	4518	**17.31**	**0.008**	0.096	
SA (age 24)	4518	**9.25**	**−0.109**	0.098	
SA (age 26)	4518	**10.49**	**− 0.109**	0.098	

*REML* restricted maximum likelihood analysis, *LDSC* linkage disequilibrium score regression, *h*^*2*^_*SNP*_ SNP-based heritability, *SE* standard error, *ADHD* attention deficit/hyperactivity disorder, *ASC* autism spectrum conditions, *NSSH* non-suicidal self-harm, *SA* suicide attempt

### Genetic correlations between NSSH, SA and related phenotypes

**Fig. 1:**
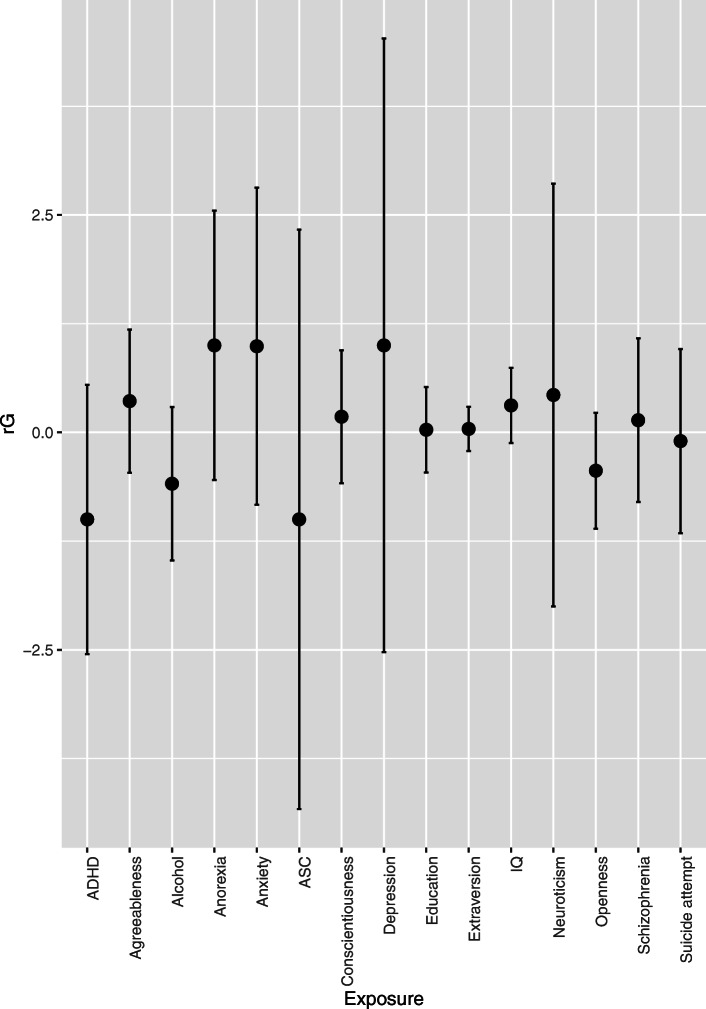
Bivariate restricted-maximum likelihood analysis showing genetic correlation between non-suicidal self-harm and related phenotypes. Notes: rG genetic correlation; error bars represent 95% confidence interval of rG estimate. ADHD attention deficit/hyperactivity disorder; ASC Autism spectrum conditions. Confidence intervals for income and cannabis use were extremely wide and so are not shown

As SNP heritability for SA was negligible, genetic correlations were only estimated between NSSH and related phenotypes (Fig. 1, Supplementary Table 1). No exposure trait had robust evidence of correlation with NSSH: all rG estimate 95% confidence intervals (CIs) crossed the null. Of all the related phenotypes, IQ had the strongest evidence for correlation with NSSH (rG = 0.31 SE = 0.22 *p* = 0.08), and NSSH showed little evidence of correlation with the SA measure at up to age 26 (rG = − 0.10 SE 0.54 *p* = 0.43).

### Differential association and prediction of NSSH and SA using polygenic risk scores

In the multinomial regression models (Table 3 and Supplementary Figure 1), the standardised PRS for ADHD, ASC, depression, neuroticism, agreeableness and suicide attempt were associated with both NSSH and SA. There was also tentative evidence for an association between the PRS for schizophrenia and both outcomes. Depression was the only predictor that was differentially associated with NSSH and SA whereby associations were markedly stronger for SA than NSSH (NSSH RR 1.17, 95% CI 1.08, 1.27; SA RR 1.47, 95% CI 1.32, 1.63). For some PRS associations were found for one behaviour only (although CIs overlapped). For example, PRS for IQ, alcohol consumption and education were associated with NSSH but not SA, whereas PRS for anorexia and lower income were associated with SA but not NSSH. We found no strong evidence of associations between PRS for anxiety, cannabis use, conscientiousness, extraversion, or openness with either outcome.

**Table 3:** Multinomial Poisson logistic regression results (*n* = 4959) showing association between polygenic risk score for 17 related phenotypes with non-suicidal self-harm and suicide attempt

	Non-suicidal self-harm		Suicide attempt	
Exposure	RR	95% CI	RR	95% CI
ADHD	1.10	1.02, 1.19	1.21	1.09, 1.34
Alcohol	1.07	0.98, 1.16	0.99	0.89, 1.10
Anorexia	1.01	0.93, 1.09	1.12	1.01, 1.23
Anxiety	0.98	0.90, 1.05	0.96	0.87, 1.06
ASC	1.09	1.01, 1.18	1.09	0.98, 1.20
Cannabis	1.03	0.95, 1.11	0.99	0.89, 1.09
Conscientiousness	1.02	0.94, 1.10	1.00	0.90, 1.11
Depression	1.17	1.08, 1.27	1.47	1.32, 1.63
Education	1.04	0.96, 1.12	0.97	0.88, 1.07
Extraversion	0.98	0.91, 1.06	0.97	0.88, 1.08
Income	1.01	0.93, 1.09	0.90	0.81, 0.99
IQ	1.14	1.06, 1.24	1.02	0.92, 1.13
Neuroticism	1.16	1.08, 1.26	1.27	1.14, 1.40
Agreeableness	0.94	0.87, 1.02	0.93	0.84, 1.03
Schizophrenia	1.05	0.97, 1.14	1.06	0.96, 1.17
Openness	1.01	0.93, 1.09	1.02	0.92, 1.13
Suicide attempt	1.07	0.99, 1.15	1.10	0.99, 1.21

Reference category: no self-harm. Polygenic scores standardised to have mean 0 and standard deviation 1 so the RR represents the change in risk of the outcome per 1 standard deviation increase in polygenic risk score

*ADHD* attention deficit/hyperactivity disorder, *ASC* autism spectrum conditions

**Fig. 2:**
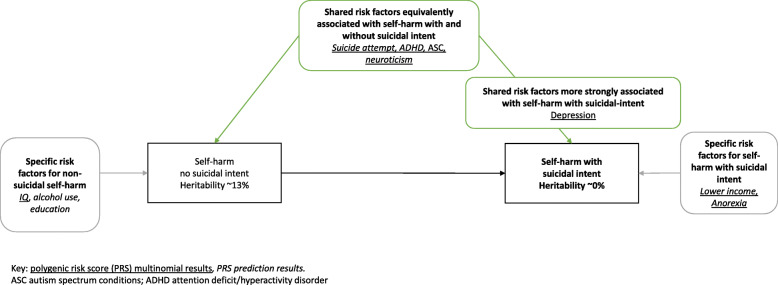
Summary of findings

The optimal prediction model for SA included PRSs for ADHD, depression, income, anorexia and neuroticism. The model had a Nagelkerke R^2^ of 0.0222 (*p* < 0.001), with 2.22% of the variance explained by the measured PRSs. This was higher than Nagelkerke R^2^ calculated from the model including the PRS for SA alone (Nagelkerke *R*^2^ = 0.12%). The optimal prediction model for NSSH had a Nagelkerke R^2^ of 0.0104 (*p* < 0.001). This model included ADHD, alcohol consumption, ASC, depression, IQ, neuroticism and suicide attempt (Supplementary Table 2). Betas were much larger for SA than NSSH for both ADHD and depression. Supplementary Table 3 shows the model fit statistics for optimal models. The findings from the PRS multinomial and prediction models are summarised in Fig. 2.

## Discussion

We explored the genetic architecture and overlap between non-suicidal self-harm and suicide attempt using detailed phenotypic and genotype data from the ALSPAC cohort. Our findings suggest that the variance captured by SNPs is small, and there was a greater proportion of common genetic variation explained for NSSH than SA. These heritability estimates were lower than for personality and sociodemographic traits such as alcohol consumption, but similar to other psychiatric phenotypes in our sample. Twin studies have estimated the heritability of suicidal behaviours to be between 17 and 48% [7]. The SNP-based heritability estimate for SA was in line with other studies [8, 9], and although our estimate increased to 6% when including an extra 2 years of data, standard errors were large. There are several potential explanations for this: the sample with SA at 26 years includes an additional 131 individuals with a history of SA compared with age 24 years, which may have provided additional statistical power to calculate heritability. It may also be that there are differences in the aetiology of SA between adolescence and young adulthood. Future studies should examine whether there is evidence to support this.

The small genetic correlations indicated little overlap between the genetic architectures of NSSH and related phenotypes, supporting evidence that NSSH does not solely occur in the context of psychiatric disorder [39]. Although we were unable to explore genetic correlations for related phenotypes with SA in ALSPAC due to its negligible heritability, we found little evidence of correlation between NSSH and SA measured up to age 26 (matching the external GWAS), suggesting they share little genetic overlap. However, our findings must be considered alongside acknowledgement of our relatively small sample size and large standard errors around our rG estimates; sampling error may outweigh any signal. This particularly applies to schizophrenia, problematic cannabis use, and anorexia, where the prevalence of each was very low in ALSPAC. This would affect bREML analyses but not PRS as those scores were derived using external GWAS data.

Our estimates of shared heritability between our related phenotypes and NSSH are smaller than those of a similar study that calculated genetic overlap between psychiatric traits, (although not NSSH or SA) that reported correlations between phenotypes of up to 50% [40]; further research with larger sample sizes is needed in order to confirm whether NSSH has relatively weaker genetic correlations with psychiatric disorders than other traits. If our finding is replicated, this implies that genetic risk for NSSH may be independent of genetic risk for other psychopathologies.

Prior analysis of observational data in ALSPAC found IQ and maternal education were differentially associated (opposite directions) with NSSH and SA [13]. Depression, anxiety and substance dependence were found to have stronger associations with SA than NSSH (although were associated with both) [13]. In the current study, we found an association between the PGS for IQ and NSSH but not for SA (although the CIs overlapped) and IQ showed the strongest evidence of a genetic correlation with NSSH. Low IQ has been associated with suicide and SA in several prior studies [41–43] but the association with NSSH has been relatively unexplored.

Our phenotypes of self-harm and suicide attempt were restricted to early onset suicidal behaviour, as the cohort was age 26 at the final measurement point. The impacts of this may mean that we underestimated differences between those with NSSH and SA. Self-harm is most prevalent in younger age groups and frequently has an onset before the age of 17 [44, 45], however suicide attempt may have a much later age of onset; a study in 2012 found that over 30% of suicide attempts and other suicidal behaviour were initiated over the age of 30 years [46]; another study found a median age of onset of suicidal behaviour of 55 years, and different factors were associated with early and late-onset suicidality. For example, earlier-onset suicidal behaviour has been associated with negative life events, maladaptive personality traits, more difficulty regulating behaviour and planning ahead, whereas later-onset suicide attempt (after age 55) appears to be linked more closely to proximal stressful life events [47]. Our findings therefore cannot be generalised into older populations without further investigation.

The findings from our multinomial regression provide tentative evidence that some risk factors for NSSH and SA are shared, whereas others may be specific, although the standard errors of many of our estimates were very large. Depression was the only shared risk factor that was clearly more strongly associated with SA than NSSH. This replicates previous observational findings [8, 14, 48–50]. However, other previously-reported observational associations were not replicated in our analysis. For example, we did not find evidence for an association between the PRS for anxiety and either outcome, whereas some prior studies have found anxiety to be more strongly related to SA than to NSSH [14, 48, 51]. In addition, neuroticism has been found to be more strongly associated with SA in observational studies [52], and with SA but not NSSH in a prior PRS study [18] whereas we found it to be similarly associated with both outcomes.

### Strengths and limitations

ALSPAC is a population-based birth cohort that is broadly representative of the general UK population. Detailed phenotypic information was available on a wide range of exposures and data were collected prospectively and often using validated measures (see Table 1). Prevalence estimates of NSSH were 17.0% and SA 9.5% in our sample which is in line with other epidemiological studies [53]. It should be noted that these can be considered “early onset” suicidal behaviour, given that age 26 years was our latest measure of self-harm, and therefore our findings may not generalise to apply to suicidal behaviour across the life course. Terminology and definitions of self-harm are complex and vary across countries and individual studies. In the UK, self-harm is commonly used as an umbrella term to capture self-injurious acts regardless of intent [54], and in this study we have defined suicide attempt as being a history of self-harm with self-reported intent to die in at least one self-harm event.

The clinical reality is more complex, and those who self-harm may vary in their intent between episodes as well as over time, as such our approach may be reductionist and prone to potential reporting difficulties. In addition, different methods of self-harm (which themselves are patterned by gender) are differentially associated with suicidal intent. Completed suicide is associated with previous suicidal intent for the majority of individuals, but not all [54]. A more nuanced understanding of self-harm and comprehensive assessment of the nature, purpose and intent of self-harm is needed in future studies to overcome these limitations.

Our estimates of NSSH and SA are substantially higher than those reported in UK BioBank (a large, convenience, socially-advantaged sample) [8, 19], and these sample and phenotypic differences may explain differences between our PRS findings and recent UK BioBank studies [8, 18]. The largest current GWAS of SA outside of UK BioBank [9] used psychiatric case records to identify a large number of cases which increased power, however the sample is therefore not representative of suicidal behaviour in the general population as many had severe mental disorders. This may explain why we did not find an association between the PRS for SA derived from this previous GWAS and SA in ALSPAC.

Because the genetic basis of self-harm and suicidal behaviour is still poorly understood, we cannot be sure if we failed to find associations because there really is little genetic basis to these traits, or because of a lack of statistical power. Increased prediction power may become achievable in the future as larger suicidal behaviour GWASs become available through international collaboration such as that being led by the PGC [55]. The differences between our heritability estimates for SA between using data collected at age 24 and age 26 in ALSPAC highlights the limitations of sample size on inferring genetic heritability, and our analysis should be considered exploratory in light of this.

In addition, we have defined NSSH and SA as binary outcomes from self-reported data, however in actuality these acts result from complex series of events and psychological states, and our approach may be too reductionist [53]. There are limitations in the way that we have measured and classified self-harm and suicide attempt, as we do not capture the full spectrum of what is considered to be suicidal behaviour [10, 11]. In this study we have characterised NSSH and SA as discrete behaviours, although the form in which the data were collected required report of any self-harm are a pre-requisite for participants to be asked about self-harm with suicidal intent. We did not capture other forms of suicidal behaviour in this data, such as suicidal thoughts and our data do not capture the possibility of ‘suicidality’ without self-harm.

A further limitation of our study relates to the GWAS summary statistics, from which we derived our PRS scores. The sample sizes of GWASs affects their power to detect associations with the exposures of interest, and we note that we see the largest effect sizes corresponding to the largest GWAS sample (depression), and null effects for anxiety, which had the smallest GWAS sample (~ 21,000 participants). Caution is therefore required when interpreting results from PRS. This highlights how unreliable it may be to differentiate between the relative aetiological overlaps between different traits using PRS, because the results are largely driven by power. With increasing sample sizes, these methods will become more powerful and our ability to detect associations will improve. Furthermore, although PRS are useful for generating prediction models, effect sizes tend to be very small as SNP-level variation explains only a small proportion of the variation in the outcome.

Although genetic methods control for some problems of confounding compared with using observational data, these methods are still liable to bias through, for example, assortative mating, dynastic effects and population stratification [56, 57]. Our PRS prediction model performed better for SA than NSSH, in spite of our finding little evidence of SNP-based heritability of SA. This suggests that the GWASs are potentially affected by residual confounding. Although we controlled for the first 10 principal components, this appears to be insufficient to fully capture residual confounding in this case. In practice this means our PRS prediction models are over-estimating the variance explained. Finally, we included several sociodemographic traits in our study, however it is important to note that ‘higher income’ or ‘having a degree’ are unlikely to be directly related to specific genetic variants and these pathways are therefore likely to be mediated by other characteristics and personality traits, for example attention regulation or cognitive abilities. Our alcohol exposure measure was based on self-reported frequency and consumption of alcohol on an average weekly basis, which may not tap-in to problematic alcohol use or binge drinking patterns, and similar limitations apply to other phenotypes such as cannabis use.

### Implications

Our findings add to a growing body of science focussed on understanding the genetic influences on self-harm and suicidal behaviour. We found that NSSH and SA share some genetic risk factors, including polygenic risk for SA derived from a separate GWAS, but also that some factors (i.e. depression) may be more strongly associated with SA than NSSH. From prediction models, lower income and anorexia appeared to uniquely predict SA, and higher IQ and education uniquely predicted NSSH. Taken together, our findings suggest that NSSH and SA are not simply a continuum of increasingly severe suicidal behaviour, or discrete unrelated outcomes: we found evidence that although NSSH and SA are not categorically distinct outcomes there appears to be little genetic overlap between the two.

If our findings are replicated, they can be used to inform preventative interventions for young people at risk of NSSH or SA. It would be of interest to explore whether our findings reflect identifiable typologies, and whether individuals who have several high-risk traits have common intermediary pathways that put them at a higher risk of NSSH or SA. For example, those with high IQ and high conscientiousness may also have high levels of traits such as perfectionism, which is associated with psychological distress. Poor coping strategies for managing this distress may result in NSSH. Some evidence also suggests that patterns of risk factors may be different in the presence of specific disorders and this needs to be better understood; for example one study has shown that in the presence of psychosis (a symptom of schizophrenia), low IQ is not a risk factor for SA [58]. Further genetic and observational epidemiological studies, conducted in other large cohorts that have good coverage of mental health data, are needed in order to replicate and extend our findings and to provide recommendations for practitioners and policymakers who work in the field of self-harm and suicide.

## Conclusions

We explored the genetic architecture and overlap between non-suicidal self-harm and suicide attempt using detailed phenotypic and genotype data from the ALSPAC cohort. Our findings suggest that the variance captured by SNPs is small, and there was a greater proportion of common genetic variation explained for NSSH than SA. Our findings suggest that SA does not have a large genetic component, and that although NSSH and SA are not discrete outcomes there appears to be little genetic overlap between the two.

## Supplementary Information

**Additional file 1:** **Supplementary methods.** Quality control of GWAS data in ALSPAC. **Supplementary Table 1.** Genetic correlation (rG) between exposure phenotypes and non-suicidal self-harm in ALSPAC using bivariate restricted maximum likelihood analysis. **Supplementary Table 2.** Post-estimation OLS coefficients by optimum prediction model. **Supplementary Table 3.** Summary of optimal polygenic prediction model results. **Supplementary Figure 1.** Results of multinomial regression showing relative risk of NSSH and SA by polygenic risk for 17 related phenotypes.

## Data Availability

The data that support the findings of this study are curated by 23andMe and ALSPAC, but restrictions apply to the availability of these data, which were used under license for the current study, and so are not publicly available. Data are however available from 23andMe and ALSPAC following requests from bona fide researchers. We regret that the authors of this study are unable to make the data publicly accessible.

## Abbreviations

NSSH: Non-suicidal self-harm
SA: Suicide attempt
ADHD: Attention deficit/hyperactivity disorder
IQ: Intelligence quotient
GWAS: Genome-wide association study
SNP: Single nucleotide polymorphism
PRS: Polygenic risk score
GCTA: Genome-wide complex traits analysis
(b)REML: (Bivariate) restricted maximum likelihood analysis
LDSC: Linkage disequilibrium score regression
DSM-5: Diagnostic and Statistical Manual of Mental Diseases (fifth edition)
MDD: Major depressive disorder
ASC: Autistic spectrum conditions
ALSPAC: Avon Longitudinal Study of Parents and Children
GPC: Genetics of Personality Consortium
SE: Standard error
CI: Confidence interval
RR: Relative risk/risk ratio
