# Supplementary material for: An exploration of the genetic epidemiology of non-suicidal self-harm and suicide attempt
Source: BMC Psychiatry. 2021 Apr 23;21:207. doi: 10.1186/s12888-021-03216-z (PMC8066869; doi:10.1186/s12888-021-03216-z)
Supplement: Supplementary file 1 — Additional file 1: Supplementary methods. Quality control of GWAS data in ALSPAC. Supplementary Table 1. Genetic correlation (rG) between exposure phenotypes and non-suicidal self-harm in ALSPAC using bivariate restricted maximum likelihood analysis. Supplementary Table 2. Post-estimation OLS coefficients by optimum prediction model. Supplementary Table 3. Summary of optimal polygenic prediction model results. Supplementary Figure 1. Results of multinomial regression showing relative risk of NSSH and SA by polygenic risk for 17 related phenotypes. [file 12888_2021_3216_MOESM1_ESM.pdf]

Supplementary Material for *the genetic epidemiology of non-suicidal self-harm and suicide attempt*

Supplementary methods: quality control of GWAS data in ALSPAC

The resulting raw genome-wide data were subjected to standard quality control methods. Individuals were excluded on the basis of gender mismatches; minimal or excessive heterozygosity; disproportionate levels of individual missingness (>3%) and insufficient sample replication (IBD < 0.8). Population stratification was assessed by multidimensional scaling analysis and compared with Hapmap II (release 22) European descent (CEU), Han Chinese, Japanese and Yoruba reference populations; all individuals with non-European ancestry were removed. Single nucleotide polymorphisms (SNPs) with a minor allele frequency of < 1%, a call rate of < 95% or evidence for violations of Hardy-Weinberg equilibrium (P < 5E-7) were removed. Cryptic relatedness was measured as proportion of identity by descent (IBD > 0.1). Related subjects that passed all other quality control thresholds were retained during subsequent phasing and imputation. 9,115 subjects and 500,527 SNPs passed these quality control filters. Genotype data were imputed to the HRC panel (HRC.r1.1) using the Michigan imputation server resulting in 8,237 children with genotype data. Related individuals were excluded.

Supplementary Table 1. Genetic correlation (rG) between exposure phenotypes and non-suicidal self-harm in ALSPAC using bivariate restricted maximum likelihood analysis

Exposure phenotype	n	rG	SE	p-value	fdr-adjusted p-value
ADHD	10946	-1.00	0.79	0.103	0.370
Alcohol consumption	7993	-0.59	0.45	0.095	0.370
Anorexia	10130	1.00	0.79	0.103	0.370
Anxiety	11150	0.99	0.93	0.144	0.431
ASC	12300	-1.00	1.70	0.278	0.497
Cannabis problematic use	6415	-1.00	137.09	0.497	0.497
Conscientiousness	8627	0.18	0.39	0.322	0.497
Depression	11248	1.00	1.80	0.289	0.497
Education	8513	0.03	0.25	0.452	0.497
Extraversion	8817	0.04	0.13	0.379	0.497
Income	7435	1.00	9.23	0.457	0.497
IQ	10178	0.31	0.22	0.079	0.370
Neuroticism	8690	0.43	1.24	0.364	0.497
Agreeableness	8745	0.36	0.42	0.196	0.497
Schizophrenia	7250	0.14	0.48	0.385	0.497
Openness	8729	-0.44	0.34	0.098	0.370
Suicide attempt (age 26)	9245	-0.10	0.54	0.427	0.497

Notes: N is the total who have either exposure or outcome data e.g. N for ADHD + N for NSSH=10946. SE standard error. ADHD attention deficit/hyperactivity disorder ASC autism spectrum conditions. FDR false discovery rate

Supplementary Table 2. Post-estimation OLS coefficients by optimum prediction model

	Model for NSSH	Model for SA
ADHD	53.6	60.5
Alcohol	1158.8	
Anorexia		28.9
Anxiety		
ASC	123.9	
Cannabis		
Conscientiousness		
Depression	1083.4	2865.9
Education		
Extraversion		
Income		-131.0
IQ	1264.3	
Neuroticism	8.8	3.8
Agreeableness		
Schizophrenia		
Openness		
Suicide attempt	116.5	

Supplementary Table 3. Summary of optimal polygenic prediction model results

Model	Lambda	Mean Squared Predication Error	SD
NSSH	41.968	0.142	0.003
SA	40.362	0.083	0.002

Supplementary Figure 1. Results of multinomial regression showing relative risk of NSSH and SA by polygenic risk for 17 related phenotypes

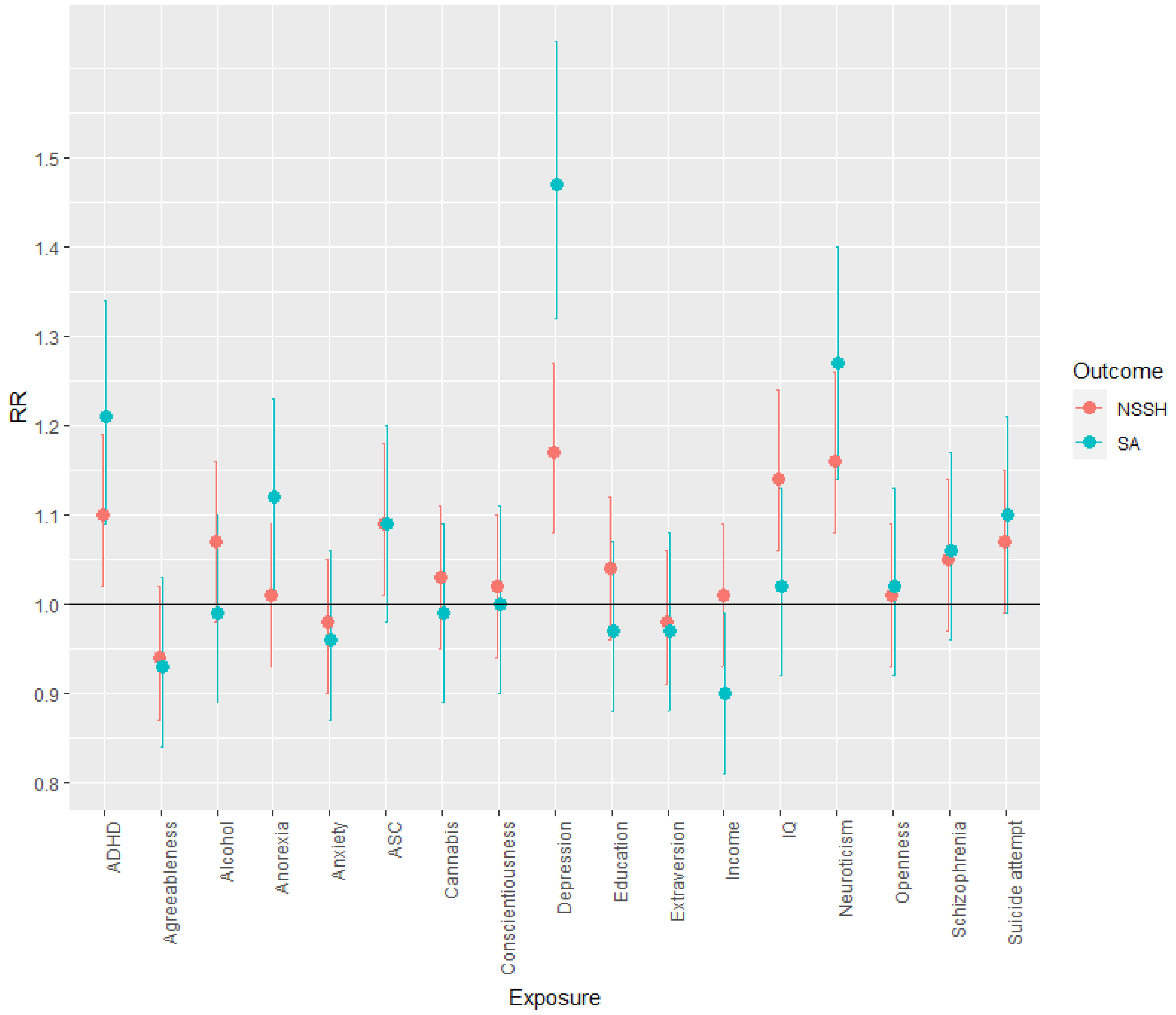

Notes: RR Relative risk; error bars show 95% confidence interval; NSSH non-suicidal self-harm; SA suicide attempt; ADHD attention deficit/hyperactivity disorder; ASC autism spectrum conditions
